# 血小板生成素受体激动剂在非肌球蛋白重链9相关疾病治疗中的应用

**DOI:** 10.3760/cma.j.cn121090-20250919-00428

**Published:** 2026-03

**Authors:** 雨 龚, 文兰 陈, 艳洁 胡, 恒 梅, 雅丹 王

**Affiliations:** 华中科技大学同济医学院附属协和医院血液病研究所，武汉 430023 Institute of Hematology, Union Hospital, Tongji Medical College, Huazhong University of Science and Technology, Wuhan 430023, China

## Abstract

非肌球蛋白重链9相关疾病（MYH9-RD）是一种由MYH9基因突变导致的以血小板减少、巨大血小板、中性粒细胞包涵体等为主要特征的常染色体显性遗传病。由于其发病率较低和临床表现缺乏特异性，易被误诊和漏诊。血小板输注、使用促止血或抗纤溶药物、避免使用抗血小板药物和预防出血等常用于该类患者的临床管理，造血干细胞移植有可能成为严重病例的治疗方法。长期以来，血小板输注是预防或治疗MYH9-RD患者出血的最常用的方法，但仍存在许多不良反应和临床应用局限性。近年来，血小板生成素受体激动剂（TPO-RA）逐渐被用于MYH9-RD的治疗，在提高患者血小板计数和改善出血症状方面展现出良好的疗效。然而，关于其机制、临床疗效及安全性仍需进一步探讨。本文综述TPO-RA在MYH9-RD中的应用，分析其作用机制、临床疗效及未来的研究方向，为该领域的研究提供参考和借鉴。

## Abstract (en)

MYH9-related disease（MYH9-RD）is an autosomal dominant disorder caused by mutations in the MYH9 gene, characterized by macrothrombocytopenia, neutrophil inclusions, and other features. Due to its low incidence and nonspecific clinical manifestations, many patients are misdiagnosed or undiagnosed. Clinical management often includes platelet transfusion, pro-hemostatic or antifibrinolytic agents, avoidance of antiplatelet drugs, and hemorrhage prevention. Hematopoietic stem cell transplantation may be a treatment option for severe cases. For a long time, platelet transfusion has been the most commonly used treatment for preventing or managing bleeding in MYH9-RD patients, but it is associated with various adverse reactions and clinical limitations. In recent years, thrombopoietin receptor agonists（TPO-RA）, as a novel therapeutic class, have been increasingly used in MYH9-RD and have demonstrated promising efficacy in increasing platelet counts and reducing bleeding symptoms. However, their mechanisms of action, clinical effectiveness, and safety profiles require further investigation. This article aims to review the application of TPO-RA in MYH9-RD, analyze their mechanisms, clinical outcomes, and future research directions, thereby providing insights and references for further studies in this field.

非肌球蛋白重链9相关疾病（MYH9-related disease, MYH9-RD）是由MYH9基因突变引起的常染色体显性遗传性血小板减少症[1]–[2]。该基因编码非肌性肌球蛋白重链ⅡA（NMMHC-ⅡA），其功能缺陷导致巨核细胞胞质分裂障碍，表现为巨大血小板伴血小板减少、中性粒细胞包涵体；除血液系统表现外，30％～50％的患者随年龄增长出现进行性肾小球肾炎、感音性耳聋或早发性白内障等多系统损害[3]。MYH9-RD是遗传性血小板减少症中最常见的亚型，占确诊患者的20％～25％，其预估发病率为3.75/1 000 000（意大利人群数据），但因表型异质性和漏诊，实际可能更高[1]。患者自出生即存在巨大血小板及血小板减少症，但其严重程度存在显著的基因型-表型相关性。位于NMMHC-ⅡA头部结构域的突变（如R702、S96）往往导致更严重的血小板减少和更显著的出血倾向，同时伴随更高的肾脏损害、听力损失及白内障发生风险[4]–[5]。相比之下，位于尾部结构域的突变（如D1848、R1933）则表现相对温和，血小板减少程度较轻，且非血液系统并发症的发生率和严重程度均较低[6]。作为一种遗传性疾病，目前尚无根治的方法，对该类患者的出血管理提倡采取分层策略：无症状者仅需观察，避免使用抗血小板药物；轻度出血采用局部止血联合抗纤溶药物；而对于严重出血或需要手术或有创操作的患者，血小板输注仍是快速提升PLT的常规治疗方案[7]。然而，这一传统策略存在明显局限性，包括疗效被动、血小板供应不稳定，以及输血相关过敏反应、感染风险和同种免疫反应等安全隐患。特别是在临床实践中，常面临患者既存在明显出血倾向，又有严重血小板输注过敏史的困境[8]。因此，亟需能够主动、有效提升PLT且减少输注依赖的更优治疗方案。

血小板生成素受体激动剂（TPO-RA）通过激活c-MPL/JAK2/STAT5通路促进巨核细胞增殖和血小板生成，其疗效已在免疫性血小板减少症（ITP）、再生障碍性贫血（AA）和肝病相关血小板减少症等疾病中得到证实[9]–[10]。近年来，TPO-RA在MYH9-RD中的潜在价值逐渐显现。本文将结合国内外研究报道和本中心应用经验，系统综述TPO-RA在MYH9-RD中的分子机制、临床疗效及安全性证据，以期为临床实践提供循证依据。

一、MYH9-RD的发病机制

MYH9-RD的发病机制主要源于MYH9基因突变导致的NMMHC-ⅡA功能异常[11]。NMMHC-ⅡA作为细胞骨架的重要组成部分，在巨核细胞发育和血小板生成过程中发挥着关键作用[12]。在正常生理状态下，造血干细胞经过增殖分化形成巨核细胞，这些细胞成熟后会发生显著的细胞质重塑，经历前血小板形成（Proplatelet Formation, PPF）过程，形成多个长而分支的突起，即前血小板，这些前血小板最终从其尖端释放出成熟的血小板[12]–[13]。NMMHC-ⅡA通过调节前血小板突起的反复弯曲和分支，增加前血小板末端的数量，从而促进血小板的正常释放[13]–[15]。然而，在MYH9-RD患者中，由于NMMHC-ⅡA功能异常，巨核细胞中前血小板的结构发生显著改变，表现为分支复杂性降低且尖端膨大，最终导致血小板数量减少和体积增大的特征性表现[11],[13]（图1）。

**图1:**
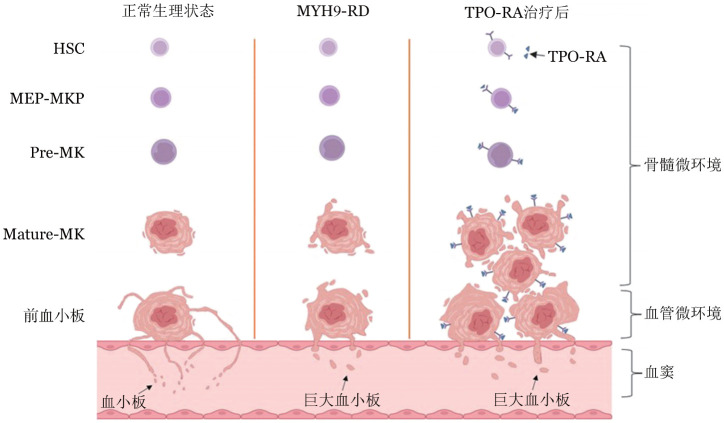
MYH9-RD发病机制及TPO-RA治疗作用模式图 **注** MYH9-RD：非肌球蛋白重链9相关疾病；TPO-RA：血小板生成素受体激动剂；HSC：造血干细胞；MEP-MKP：巨核系祖细胞；Pre-MK：前巨核细胞；Mature-MK：成熟巨核细胞

巨核细胞的成熟和血小板生成是一个高度协调的空间调控过程。在骨髓微环境中，巨核细胞首先在成骨微环境（osteoblastic niche）完成分化、增殖和成熟，随后迁移至富含窦状体的血管微环境（vascular niche），这是前血小板的生理部位[14]。NMMHC-ⅡA在这一过程中发挥着重要的负调控作用，它通过介导巨核细胞膜表面GPⅠa/Ⅱa与Ⅰ型胶原的黏附，抑制巨核细胞在成骨细胞微环境中过早形成前血小板[16]–[19]。然而，在MYH9-RD患者中，这一精细的调控机制被破坏，导致巨核细胞在错误的位置过早释放前血小板，造成血小板生成效率低下[11]（图1）。此外，巨核细胞对基质细胞衍生因子1（SDF-1）驱动的迁移效应受损，进一步加重前血小板异位释放[20]。

这些病理改变共同解释了MYH9-RD患者典型的血液学表现：血小板减少伴巨大血小板症。值得注意的是，NMMHC-ⅡA的功能异常不仅影响造血系统，还会累及其他表达该蛋白的组织和器官，如肾脏、内耳和眼[21]–[23]，这为理解MYH9-RD的多系统临床表现提供了分子基础。

二、MYH9-RD的出血机制

MYH9-RD患者多表现为轻至中度的出血症状，研究表明其自发性出血严重程度与PLT呈显著负相关（*P*<0.001），提示血小板数量是决定出血风险的关键因素之一[6]。在血小板功能方面，尽管多数临床观察显示患者体外血小板聚集功能正常或仅轻度受损[24]–[25]，但Canobbio等[26]研究揭示了更深层的功能缺陷：MYH9突变会导致依赖于细胞骨架的血小板止血功能障碍，具体表现为：在静息状态下，血小板细胞骨架已处于部分激活状态，丧失了正常的静息生理特征；在激动剂刺激后，整合素αⅡbβ3和α2β1、酪氨酸激酶及小GTP酶等关键信号蛋白与细胞骨架的结合发生障碍，导致血小板无法完成正常的形态改变、伪足形成及在血管损伤处的铺展（spreading）过程。这些本质缺陷使得患者血小板虽然在体外能正常聚集，但在体内复杂的血流动力学环境和内皮相互作用中稳定性显著下降，最终导致止血功能受损。值得注意的是，血小板骨架的异常也可能通过促进血小板活化，来部分代偿因血小板数量减少带来的出血风险。综上所述，MYH9-RD患者的出血机制是血小板数量减少与质量异常共同作用的结果，而巨大血小板在复杂生理环境中的真实功能状态及其对止血效应的综合影响仍需更深入的研究。

三、TPO-RA在MYH9-RD中的作用机制

TPO-RA可分为小分子非肽（艾曲泊帕、海曲泊帕、阿伐曲泊帕和芦曲泊帕）和小分子拟肽（罗普司亭）两大类[27]。其通过特异性结合并激活造血干细胞和各个分化阶段的巨核细胞表面的血小板生成素受体（TPO-R，又称c-MPL），模拟内源性血小板生成素（TPO）的生理作用[28]–[29]。与TPO类似，TPO-RA通过激活JAK2/STAT5、PI3K/AKT和MAPK/ERK等下游信号通路，促进巨核细胞的增殖、分化和成熟[30]–[31]。

在MYH9-RD中，致病突变主要影响NMMHC-ⅡA介导的PPF和血小板释放过程（即血小板生成缺陷），而非巨核细胞的分化与成熟[6],[32]。研究表明，MYH9-RD患者的巨核细胞数量正常甚至增多，TPO-R表达和功能完整，且在体外对TPO刺激表现出良好应答[3],[11],[33]。这一病理特点为TPO-RA的应用提供了理论基础：通过增强巨核细胞的增殖和分化能力、提高成熟巨核细胞数量，可能部分代偿NMMHC-ⅡA缺陷导致的血小板生成障碍（图1）。后续将TPO-RA应用于MYH9-RD的临床实践，进一步证实这一假说。

四、TPO-RA在MYH9-RD中的临床应用

1. TPO-RA应用于MYH9-RD的相关临床研究：目前，TPO-RA在多种获得性血小板减少症的临床应用中已积累了丰富的循证医学证据[34]–[39]。然而，关于TPO-RA治疗MYH9-RD的研究数据仍相对有限，这主要归因于该疾病的罕见性，导致临床试验难以开展。迄今为止，仅在欧洲开展了两项前瞻性临床研究，其结果均表明TPO-RA能够显著提升MYH9-RD患者的PLT，并有效改善出血倾向[24]–[25]。

2010年，首项关于艾曲泊帕治疗MYH9-RD的Ⅱ期多中心临床研究奠定了TPO-RA在该领域应用的基础[25]。研究纳入12例基线PLT<50×10^9^/L（显微镜人工计数法）的MYH9-RD患者，以剂量递增方案（50～75 mg/d）治疗42 d。治疗第21天时，42％（5/12）的患者获得主要应答（PLT>100×10^9^/L或至少升至治疗开始时PLT的3倍），33％（4/12）获得次要应答（升高为基线时的2倍）；至第42天，主要应答率升至67％（8/12），仅1例未获任何应答（该例为p.S96L突变型），平均PLT由基线31×10^9^/L升至104×10^9^/L（*P*＝0.002）。11例获得主要和次要应答的患者停药15 d后平均PLT仍高于基线值，30 d后恢复至接近基线的水平。80％（8/10）的基线出血的患者（治疗前WHO出血评分为1～2级）症状完全消失。4例脾切除患者的平均PLT显著高于未切脾患者（151×10^9^/L对81×10^9^/L），提示脾切除可能增强TPO-RA的疗效。突变位点p.R702C和p.S96L对艾曲泊帕的治疗反应较差，但因病例数太少无法明确各种基因型与治疗疗效间的相关性。

随后，欧洲学者开展了一项涵盖5种遗传性血小板减少症（IT）的Ⅱ期多中心、前瞻性研究[24]，其中纳入9例MYH9-RD患者，均接受3～6周50～75 mg/d艾曲泊帕治疗，采用显微镜人工计数法计数PLT。最终，77.8％（7/9）的患者获得主要应答（PLT>100×10^9^/L），22.2％（2/9）获得次要应答（PLT至少为基线时的2倍），平均PLT增加了98.1×10^9^/L。所有（3/3）基线存在自发出血的患者（治疗前WHO出血评分为2～3级）症状完全缓解，证实了TPO-RA在改善临床出血终点上的价值。2例临床上明显自发性出血（瘀斑、齿龈出血、鼻出血、月经量增多）的患者进入额外16周25～50 mg/d艾曲泊帕长期维持治疗，均获得了自发出血症状的持续缓解，与出血相关的健康相关生活质量（HR-QoL）也得到稳定改善，初步显示通过长期服用相对低剂量的艾曲泊帕即可维持临床获益。另外值得注意的是，相较于其他类型的IT，艾曲泊帕对MYH9-RD的PLT提升效果最好。

2. TPO-RA应用于MYH9-RD的相关临床实践：近15年来，随着对MYH9-RD认识的深入及新型TPO-RA的研发进展，TPO-RA在该疾病治疗中的地位日益凸显。多项临床实践证实，多种TPO-RA不仅能显著提升MYH9-RD患者的PLT、改善出血症状，更在围手术期管理中展现出独特优势，为需要接受外科手术或有创操作的患者提供了新的治疗选择[8],[24]–[25],[40]–[57]（表1）。

**表1:** TPO-RA在MYH9-RD中的临床应用

参考文献	发表年份	国家	报告例数	突变区域/位点	性别	年龄（岁）	操作/手术	药物	剂量	基线PLT（×10^9^/L）	治疗时间	治疗后PLT（×10^9^/L）
Pecci等[25]	2010	意大利	12	头部结构域4例，尾部结构域8例	男5例，女7例	35±14.3	–	艾曲泊帕	50～75 mg/d	31.2±14.8	3～6周	104.7±56.7
Gröpper等[42]	2012	德国	1	不详	男	30	–	罗普司亭	每周1～10 µg/kg	7	7个月	66
Pecci等[43]	2012	意大利	1	p.D1424H	女	40	–	艾曲泊帕	50 mg/d	19	17 d	195
Favier等[40]	2013	法国	1	p.S96L	女	13	鼓膜成形术	艾曲泊帕	25～50 mg/d	10	4周	70
Yamanouchi等[41]	2015	日本	1	p.Q1836R	女	42	开颅手术	罗普司亭	每周1～5 µg/kg	25	6周	84
Rabbolini等[44]	2018	澳大利亚	1	p.S96L	不详	41	–	罗普司亭	每周6.3 µg/kg	<10	41个月	超过基数值3倍，但<100
Favier等[8]	2018	法国	1	p.M1934W	女	41	剖宫产	艾曲泊帕	50 mg/d	30	19 d	179
Conte等[45]	2018	美国	1	p.D1424V	女	51	子宫切除术	艾曲泊帕	50 mg/d	21	4周	130
Zaninetti等[46]	2019	意大利	5	p.D1424H	女	40 41 43	骨科手术 骨科手术 肾穿刺	艾曲泊帕 艾曲泊帕 艾曲泊帕	50 mg/d 50 mg/d 50 mg/d	19 20 23	20 d 20 d 21 d	180 172 161
				p.R1165L	女	52 54	腹腔镜子宫切除术 人工耳蜗植入	艾曲泊帕 艾曲泊帕	75 mg/d 75 mg/d	15 17	21 d 22 d	75 78
				p.N93K	女	44 47 50 53	拔牙 牙周手术 拔牙 牙周手术	艾曲泊帕 艾曲泊帕 艾曲泊帕 艾曲泊帕	75 mg/d 75 mg/d 75 mg/d 75 mg/d	7 9 10 10	21 d 21 d 21 d 21 d	100 120 95 132
				p.D1447V	男	47	人工耳蜗植入	艾曲泊帕	75 mg/d	25	23 d	104
				p.K74del	男	49	扁桃体活检	艾曲泊帕	75 mg/d	5	21 d	11
Porrazzo等[47]	2019	意大利	1	p.R1933*	女	不详	腹腔镜卵巢切除	艾曲泊帕	50 mg/d	32	28 d	140
Mori等[53]	2022	日本	1	p.R702H	女	45	人工耳蜗植入	艾曲泊帕	小剂量（不详）	8（43岁时）	不详	>100
Zaninetti等[24]	2020	意大利	9	头部结构域3例，尾部结构域6例	男2例，女7例	42.9±14.7	–	艾曲泊帕	50～75 mg/d	38.2±22.7	3～6周	136.3±68.0
Paciullo等[48]	2020	日本	1	p.D1447V	女	72	化疗	艾曲泊帕	50～75 mg/d	20	4周	185
Arif等[49]	2022	中国	1	p.G1517V	男	24	–	艾曲泊帕 阿伐曲泊帕	50～75 mg/d 20～40 mg/d	<20 16	3个月 2周	<20 107
Lassandro等[50]	2022	意大利	1	p.Q706E	女	10	–	艾曲泊帕	25 mg/d	18	4个月	134
Nakamura等[51]	2023	日本	1	p.R702C	女	15	腹膜透析术	艾曲泊帕	25～75 mg/d	30	3周	61
Kato等[54]	2025	日本	1	p.R702C	女	19	肾移植	艾曲泊帕	50～75 mg/d	10	6周	156
Davulcu等[52]	2024	土耳其	1	p.S96L	男	27	肾脏替代透析	艾曲泊帕	50～75 mg/d	40	不详	100
Carter等[55]	2025	加拿大	1	p.R702Q	男	14	肾移植	艾曲泊帕	术前：50 mg/d 术后：血小板输注 1 U + 100 mg/d → 50 mg/d	术前：41 术后（出血时）：51	术前：33 d 术后：3个月	术前：119 术后：>100
Tan等[56]	2025	中国	1	c.2977-75C>T	男	22	–	海曲泊帕	5 mg/d	22	4周	92
孔雪婷等[57]	2025	中国	1	p.N93K	男	47	化疗	阿伐曲泊帕	40 mg/d	20～70	6个疗程的R-CHOP化疗期间	150～215

**注** TPO-RA：血小板生成素受体激动剂；MYH9-RD：非肌球蛋白重链9相关疾病；R-CHOP：利妥昔单抗+环磷酰胺+多柔比星+长春新碱+泼尼松；–：无手术

在临床应用方面，艾曲泊帕作为最早用于MYH9-RD治疗的TPO-RA，已在多种手术场景中取得显著成效。研究显示，术前3～4周开始应用艾曲泊帕（50～75 mg/d）的治疗方案，可使大多数患者在不依赖血小板输注的情况下安全完成手术，包括骨科手术、妇科腹腔镜手术、透析导管置入术、人工耳蜗植入术、肾移植等[8],[40],[46],[51]–[55]。如Zaninetti等[46]系统评估了5例重症MYH9-RD患者（基线PLT<50×10^9^/L，显微镜人工计数法）共11次手术的围手术期TPO-RA管理方案，研究采用标准化给药策略（50～75mg/d艾曲泊帕术前3周），结果显示91％手术术前达到目标PLT［（75～180）×10^9^/L］，其中1例患者在10年间4次手术均获得稳定的血小板提升效果，证实了治疗的重复性和可靠性。Paciullo等[48]报道的合并胰腺癌的MYH9-RD病例首次证实了TPO-RA在化疗支持中的价值。在儿童患者中的应用方面，自2013年首例13岁MYH9-RD患儿术前使用艾曲泊帕成功完成鼓膜成形术后[40]，后续研究进一步验证了25～50 mg/d剂量在10岁以上儿童患者中的有效性及长期（4个月）使用艾曲泊帕的可行性[50]–[51]。

相比艾曲泊帕，罗普司亭在MYH9-RD中的应用报道较少，目前仅有3例病例报告[41]–[42],[44]。这些病例多采用每周1～10 µg/kg的剂量递增方案，其中最长治疗持续时间达41个月，且未报告血栓栓塞事件及骨髓纤维化等不良反应，初步证实了长期应用的可行性[44]。2025年，中国学者首次报道了国产新型TPO-RA海曲泊帕（5 mg/d）在MYH9-RD中的应用案例，结果显示用药2周即见效，4周后PLT峰值达92×10^9^/L，提示该药物同样适合需要快速提升PLT的临床情境[56]。

在既往ITP治疗经验中，当某种TPO-RA疗效不佳时，转换治疗可能带来显著改善。本中心2022年报道1例对艾曲泊帕（75 mg/d）反应不佳的MYH9-RD患者，在转换为阿伐曲泊帕（40 mg/d）后仅2周，PLT即从16×10^9^/L显著升至107×10^9^/L[49]。虽然其确切机制尚未完全阐明，但这一现象提示不同TPO-RA可能存在疗效差异，为临床个体化治疗策略的制定提供了重要依据。

3. 基因型与治疗疗效间的相关性：由于缺乏大样本疗效研究的数据，目前尚无法确切阐明MYH9-RD特定基因型与TPO-RA疗效之间的内在关联。通过对既往文献中31例接受艾曲泊帕标准治疗（50 mg/d，3～4周）的MYH9-RD患者进行综合分析，我们观察到基因突变区域与治疗反应之间存在显著关联。在22例尾部结构域突变患者中，17例（77.3％）治疗后PLT提升至100×10^9^/L以上，而9例头部结构域突变患者中仅有3例（33.3％）达到同等疗效水平。所有3例治疗后PLT未能达到基线值2倍的患者均携带头部结构域突变（包括1例p.S96L和2例p.R702C），提示此类突变可能导致相对耐药性。值得关注的是，其中1例p.R702C患者在剂量递增至75 mg/d继续治疗3周后，PLT恢复至正常范围，表明个体化剂量调整可能改善特定突变患者的治疗反应。这些发现初步证实了突变位点对治疗疗效的影响趋势：相较于尾部突变，头部突变（尤其是p.R702C和p.S96L）患者对艾曲泊帕的标准剂量治疗反应较差。然而，受限于现有研究数量有限和样本量较小，不同种类TPO-RA针对各基因型患者的最佳起始剂量和疗效特征仍需通过大样本前瞻性研究进一步阐明。

4. 安全性分析：TPO-RA在ITP患者中的安全性数据较为完善，常见的不良反应包括头痛（5.5％～35％）、肌痛骨痛（3.7％～11.4％）、腹痛腹泻（7.0％～12.6％）等，这些反应多为1～2级且具有可逆性[58]–[64]。不同TPO-RA的肝毒性存在差异，其中艾曲泊帕和海曲泊帕导致肝功能受损和肝酶升高的发生率相对较高（10％～15％）[61],[64]–[65]。在血栓风险方面，总体发生率维持在较低水平（低于6％），但TPO-RA可促进ITP患者血小板活化，长期应用有增加动、静脉血栓事件发生的概率[58],[60]–[62],[66]。长期用药观察发现，不到10％的ITP患者应用TPO-RA期间会进展至中度以上骨髓网状纤维化（MF-2级以上），且大多数患者在停药后可获得逆转[67]–[70]。另外，用药后新发白内障或白内障恶化的发生率低于5％[61],[71]。

TPO-RA在MYH9-RD患者中的应用经验相对有限，现有临床观察安全数据表明，短期治疗的耐受性良好。约21％的患者出现1级不良事件，主要表现为轻微而短暂的头痛、骨痛、口干和湿疹等症状[24]–[25],[46]。目前尚未在MYH9-RD患者中观察到肝酶升高、血栓栓塞、骨髓纤维化或白内障等严重不良反应。然而，考虑到MYH9-RD患者本身可能出现肝酶升高、肾炎和白内障等器官功能损伤，临床医师在用药前必须结合以上情况对患者进行个体化的全面评估。鉴于长期应用TPO-RA可能带来的血栓风险以及存在骨髓纤维化风险，且一项基础研究曾显示罗普司亭可诱导MYH9基因型小鼠发生骨髓纤维化[72]，因此当前学术界主流观点认为TPO-RA不宜作为常规促血小板生成药物长期用于MYH9-RD患者。然而，在真实世界中，对于存在严重自发出血倾向（反复鼻出血、齿龈出血、月经量增多、甚至有过消化道出血）的患儿，部分父母会自行选择长期小剂量使用TPO-RA，维持PLT在较高水平，达到减少出血事件的目的。这种管理策略是否可取尚无定论，其获益和风险有待高质量研究进一步明确。

5. TPO-RA在MYH9-RD中的应用策略：为避免药物的潜在风险，同时兼顾临床治疗需求，笔者综合既往文献和个人经验提出以下应用策略：①进行血常规检测时，应与检验人员充分沟通，使用血细胞自动计数仪的光学通道检测并进行镜下复检，必要时可进行人工计数，以获得准确的PLT从而指导临床决策。②对于PLT≥30×10^9^/L、无出血表现且不从事增加出血风险工作、无增加出血风险因素（参见ITP指南[73]）的患者，可予以观察随访。③对于轻度或中度黏膜皮肤出血的控制以局部干预为主，措施包括：鼻腔填塞、对浅表伤口使用氨甲环酸浸润纱布加压包扎，使用含氨甲环酸的漱口水来控制齿龈出血。局部止血措施失败时，再考虑启动TPO-RA短期应用。④若患者有活动性出血症状（WHO评分≥2级），应提升PLT至相对安全水平，出血症状停止后逐渐停用，持续使用时间不超过半年。⑤对于拟行操作、手术或化疗等出血风险较高的患者，尤其是既往输注血小板时发生较为严重的不良反应或者输注血小板疗效不佳的患者，建议短期应用TPO-RA（术前3～4周开始应用，术后1～2周内逐渐减量至停用），以提升PLT至相对安全水平（部分临床常规操作或手术以及接受药物治疗时PLT参考值参见ITP指南[73]）。⑥对于头部突变（尤其是p.R702C和p.S96L）患者建议使用更高的起始剂量（如艾曲泊帕75 mg/d）或药效更强的TPO-RA（比如阿伐曲泊帕）以快速获得较好的治疗反应。⑦体内艾曲泊帕31％经过尿液清除，且肾功能损伤的患者艾曲泊帕的药物暴露量下降[27]，因此对于容易伴随肾功能损伤的突变亚型（如p.S96、p.R702、p.D1424H等），建议慎用艾曲泊帕。p.D1424H突变携带者白内障风险最高，应用TPO-RA时应加强对白内障风险的监测。⑧部分患者可能伴有肝功能轻度或中度损伤，该类患者需慎用艾曲泊帕和海曲泊帕，使用时密切监测肝酶和胆红素。⑨较长时间应用TPO-RA时加强对动静脉血栓、骨髓纤维化风险的监测。

6. TPO-RA对MYH9-RD血小板大小的影响：体积巨大的血小板生成是MYH9-RD主要特征之一，这一现象与NMMHC-ⅡA功能缺陷导致的巨核细胞胞质分裂障碍、PPF异常密切相关。近年来，研究者开始关注TPO-RA治疗对MYH9-RD患者血小板大小的影响，但现有研究结果存在一定异质性。在首项关于艾曲泊帕治疗MYH9-RD的临床研究中，通过计算机辅助显微镜图像分析系统测量发现，治疗期间及停药后患者的血小板直径未出现显著变化[25]。这一结果提示，艾曲泊帕可能主要通过增加血小板数量而非改变其体积来发挥治疗作用。然而，后续个案报道显示了不同的发现。一例接受罗普司亭（每周1～5 µg/kg）术前治疗的MYH9-RD患者中，研究者观察到药物选择性地促进了大血小板（直径<7 µm）的生成，而对巨大血小板（直径≥7 µm）的影响有限[41]。另一例术前接受艾曲泊帕（50～75 mg/d）治疗4周的患者，其平均血小板体积（MPV）从基线20 fl显著降至15 fl（降低25％），且这一效应在停药后逐渐逆转[47]。这些看似矛盾的结果可能源于测量方法学差异、测量时机的差异、不同MYH9突变位点对TPO-RA的反应差异以及不同TPO-RA间差异。因此，需要采用标准化测量方案和开展基础研究阐明分子机制进而深入探索。

7. TPO-RA对MYH9-RD血小板功能的影响：关于TPO-RA对MYH9-RD患者血小板功能的影响，现有两项临床研究在有限样本中进行了初步探索。2010年的临床研究评估了7例经艾曲泊帕治疗后PLT>100×10^9^/L患者的血小板聚集功能。研究采用标准血小板体外聚集试验，结果显示5例（71.4％）患者对二磷酸腺苷（ADP）、胶原和瑞斯托霉素的诱导反应正常，2例（28.6％）对ADP和胶原的反应轻度降低，提示艾曲泊帕刺激生成的血小板聚集功能基本正常或仅轻微受损[25]。这一发现在2020年的后续研究中得到验证，该研究观察到7例患者中仅2例对低浓度ADP（5 µmol/L）的反应略降低，其余患者血小板聚集功能均正常[24]。

为进一步评估血小板活化状态，研究者采用流式细胞术检测了2例患者的血小板活化标志物。通过ADP和凝血酶受体激活肽（TRAP）诱导后，检测血小板表面GPⅡb/Ⅲa活化程度及P-选择素表达水平，结果显示治疗前后血小板活化水平无显著差异[24]。这些初步数据表明，TPO-RA在提升血小板数量的同时，可能不会促进血小板活化。

然而，现有研究存在以下局限性：样本量较小、缺乏标准化的功能评估方案、未系统分析不同突变类型对血小板功能的影响。未来研究需要建立标准化的功能评估体系、在更大样本患者群体中探索基因型-功能表型的相关性、评估长期治疗对血小板功能的影响。这些深入探索将为TPO-RA在MYH9-RD中的合理应用提供更全面的循证依据。

五、TPO-RA在MYH9-RD治疗中的临床应用展望

TPO-RA为MYH9-RD患者的出血管理提供了新的策略。现有临床证据表明，这类药物能有效提升PLT、改善出血症状，在围手术期和化疗前准备等临床场景中展现出独特优势。其良好的安全性和患者依从性，使其具有广阔的临床应用前景。然而，当前研究仍存在明显局限性。首先，长期用药的安全性和有效性数据不足，特别是对骨髓纤维化、白内障、肝功能等潜在风险的长期影响尚不明确。其次，药物作用机制研究不够深入，TPO-RA如何影响MYH9突变巨核细胞的血小板生成及巨大血小板的功能变化仍需探索。再者，现有临床研究样本量普遍偏小，缺乏大样本、多中心的长期随访数据。未来研究应重点关注以下几个方向：一是开展更大规模的临床试验，建立长期随访体系；二是深入探索不同MYH9突变位点间TPO-RA疗效的差异；三是优化用药方案，平衡疗效与安全性。同时，临床应用中需建立规范的监测体系，密切观察药物不良反应。

总体而言，TPO-RA为MYH9-RD的治疗带来了新的希望，但要充分发挥其临床价值，仍需基础研究和临床实践的持续探索。通过多学科协作和精准医疗策略的推进，我们有望为这类罕见病患者提供更安全有效的治疗方案。
